# Supplementary material for: Are carbon emissions trading and green financial instruments synergistic? -Comprehensive quantitative research based on content analysis
Source: PLoS One. 2024 Mar 7;19(3):e0298601. doi: 10.1371/journal.pone.0298601 (PMC10919601; doi:10.1371/journal.pone.0298601)
Supplement: S1 Data — (ZIP) [file pone.0298601.s001.zip › Data/地级市Z_F工作报告.zip!/┴╔─■/░░╔╜╩╨/└┤╫╘╣1⁄2╓┌║┼í╛┬φ┐╦╩2╛▌═°í┐_2006.docx]

各位代表：

现在，我代表市人民政府向大会报告工作，请予审议，并请市政协各位委员和其他列席人员提出意见。

一、2005年政府工作回顾和“十五”计划完成情况

2005年是“十五”计划的最后一年。在市委的领导下，在人大的法律监督和政协的民主监督下，我们紧紧依靠全市各族人民，认真贯彻落实科学发展观，围绕振兴老工业基地、建设和谐鞍山这一主题，努力调整经济结构，推进体制机制创新，推动各项社会事业全面发展，不断提升城市综合竞争力，提高城乡人民生活水平，较好地完成了十三届人大三次会议确定的各项工作任务和“十五”计划的各项发展目标。

――综合经济实力持续增强。初步测算，2005年全市实现地区生产总值1250亿元，比上年增长16.5％，经济总量比2000年翻一番，年均增长12.9％；全口径税收达到150.7亿元，比上年增长50.6％，五年平均增长25.9％；地方财政一般预算收入完成55.2亿元，比上年增长37.4％，比2000年增长1.5倍，年均增长19.8％；全社会固定资产投资完成314.5亿元，比上年增长45.8％，五年累计完成986.7亿元，比“九五”增长1倍。鞍山在中国城市综合实力百强中从第50位跃升至第44位。

――产业结构不断优化。第二产业实现增加值776亿元，比上年增长18.4％，五年平均增长13.2％；“一个基地、三大产业”占全市规模以上工业比重由2000年86.4％提高到91.3％。鞍钢跻身世界最具竞争力钢铁企业前8位，对全市经济发展的贡献更加突出，全年实现销售收入650.1亿元，比上年增长29.7％；实缴税金96.7亿元，占全口径财政一般预算收入59.6％，其中形成地方财政一般预算收入26.3亿元，对市本级财政贡献率达到70.4％，农业实现增加值56亿元，比上年增长8.8％，五年平均增长7.4％；全年粮食产量120.9万吨，是历史上第三个高产年。第三产业实现增加值418亿元，比上年增长14.8％，五年平均增长13.2％。高新技术产业快速发展，鞍山成为国家技术创新试点市。高新区实现地区生产总值65亿元，比上年增长30％，五年平均增长27.2％。

――改革开放稳步推进。全年推进了104户国有企业改制，对79户企业依法实施了破产，基本完成了地方国有资本从一般竞争性领域退出任务，非公有制经济占全市经济的比重上升到48％，全市基本形成国有经济以鞍钢为主、地方经济以非公有制为主的所有制经济格局。在东北地区率先建立了公共行政服务体系，基本建立起比较完善的社会保障体系。全年实际利用外资2.8亿美元，比上年增长47.4％，五年累计完成10.1亿美元，比“九五”增长2.8倍。外贸出口总额13.2亿美元，比上年增长31.6％，五年累计完成33.9亿美元，比“九五”增长83.9％。

――人居环境明显改善。新增城市绿地73万平方米，五年累计新增665万平方米。城市绿化覆盖率达到36.3％，比2000年提高了3.8个百分点。全年建成区空气环境质量有284天达到国家二级标准，比2000年增加138天。五年累计投资156亿元，建成了五一立交桥等城乡基础设施。完成住宅建设160万平方米，五年累计完成775万平方米。完成居民小区改造52个，五年累计完成238个。完成240公里农村道路改造，提前三年实现了“乡通油路”。鞍山成为全国畅通工程一等模范管理三连冠城市和国家卫生城市。

――社会事业全面进步。新建、扩建了新一中、海城高中和岫岩高中，全市人均受教育年限达到10．9年，比“九五”末提高2年。鞍山科技大学获得博士学位授予权。市救援中心全面启用。防控禽流感实现无疫区、零病例。新型农村合作医疗参合农民达到50万人，有6.3万人直接受益。城乡群众文化生活日益丰富。成功举办了省九运会。全民健身活动继续保持全国先进水平。社区“一站四室”全部达到省规定标准。群众性精神文明创建活动不断深入，鞍钢公司、西柳镇被评为全国文明单位和全国文明镇。鞍山被评为全国创建文明城市工作先进市，获得全国双拥模范城市三连冠和全国民族团结进步模范市称号。

――人民生活水平进一步提高。完成实名制就业15万人次，五年累计安置53万人次。城乡低保提标扩面，不断推进应保尽保。城市和农村住房人均建筑面积分别达到20.7平方米和20.5平方米。全年城市居民人均可支配收入达到9463元，农民人均纯收入达到4750元，比上年分别增长14.5％和11.7％，比2000年分别净增3410元和1154元，年均分别增长9.3％和5.7％。

过去的一年，我们主要做了以下九项工作：

（一）坚持工业强市方略，加快推进“一个基地、三大产业”建设。紧紧抓住项目这个核心，新建续建亿元以上项目138个，当年完成投资110亿元。海城纺织工业园翔鹭化纤、鞍千矿业、南亚实德PVC管材等33个项目竣工投产。开展工业升级年活动，完成6户企业“退二进三”搬迁改造。鞍钢牌铁路用钢轨和西洋集团西洋牌三元复合肥成为中国名牌产品，“海诺”和“衡”牌成为中国驰名商标。

（二）全力支持鞍钢发展，积极营造鞍钢经济圈。落实2.5亿元专项资金，支持鞍钢项目建设。鞍钢西部500万吨精品钢材相关项目陆续竣工，代表鞍钢自主集成技术的2150ASP热连轧生产线热负荷试车一次成功，具有世界一流水平的新二号高炉开炉出铁，从炼铁到热轧生产线全线贯通。全面推进地企合作，鼓励鞍钢项目落户地方，冀东水泥二期工程等重点项目顺利实施。

（三）稳定农业基础地位，大力推进农业产业化。全面完成第二轮土地延包工作。全面取消农业税，当年免征4600万元。发放“三补”资金5904万元。农民人均政策性增收61元。投入农业产业化专项资金1000万元，支持建设了岫岩永安滑子蘑深加工、台安仁泰肉禽、海城三合肉禽等一批农业产业化项目，发展省级以上农业产业化重点龙头企业5家。省级无公害农产品基地达到72个。农民经济合作组织增加到234个，农民经纪人超过4000人。

（四）积极发展现代服务业，加速第三产业发展。开工建设了北方五金机电城等33个旅游商贸物流项目，大福源超市等5个项目竣工营业。积极发展新型流通业态，全年新增规模以上连锁店铺65家。海城、台安被列为全国首批“万村千乡”工程试点县。加入东北“4＋1”城市区域旅游联合体。千山获得全国首批文明风景旅游区称号。全年接待境内外旅游者776.8万人次，实现旅游综合收入57.3亿元，比上年增长15％。

（五）坚持以开放促改革、促调整、促发展，推进体制机制创新。当年引进亿元以上投资项目22个。完成工程承包和劳务合作合同额5500万美元，外派劳务5000人次。加大了对台经贸工作力度。积极推进一工、北铸等10个重大改制重组项目。落实非公有制经济发展专项资金2850万元。与国家开发银行辽宁省分行合作建立融资平台，解决中小企业融资难问题取得突破。开展了农村信用社改革试点工作。

（六）加强资源保护和环境治理，着力改善城乡面貌。加大矿业秩序整顿力度，查处违法违规案件51起，依法炸毁违法违规坑口24处，取缔无证开采和环境污染选矿企业26家。全面启动南沙河综合整治工程。鞍钢被确定为国家循环经济试点单位。完成16项大气和水污染治理重点工程，取缔建成区4吨以下燃煤供暖锅炉141台。实施100公顷生物抑尘工程。矿山复垦112公顷。植树造林9.2万亩，植树2917万株。对医疗垃圾实行了无害化处理。完成了千山路隧道北洞拓宽、杨柳河支线明沟改暗渠、鞍海路和千山东路大修、鞍羊路改造等工程。

（七）全面发展社会事业，推进和谐鞍山建设。新建5所农村九年一贯制学校。在海城部分乡镇和台安、岫岩开展新型农村合作医疗试点。人民来访接待中心投入使用。继续开展“平安钢都”建设，有力维护了社会稳定。加强了安全生产和安全防火工作，有效防止了重特大安全事故发生。民族、宗教、侨务政策得到全面落实。强化了残疾人在康复、教育、就业和生活保障等方面工作。《今日关注》和《今晚会见》成为政府与群众沟通的桥梁。维护妇女儿童合法权益，促进了妇女儿童事业发展。人口与计划生育工作走在全国前列。老龄工作在创新中发展。《鞍山年鉴》获得“中国年鉴奖”。在全省率先启动了村务公开档案管理工作。鞍山获得全国人防工作先进城市称号。民兵预备役工作继续强化。气象服务、地震监测和接待服务工作达到省内一流水平。

（八）坚持以人为本，切实维护群众切身利益。优先做好就业再就业工作，推广“1＋X”就业模式，培训就业率达到61％。最低工资标准从每月290元提高到400元。社会养老、医疗、失业保险参保人数分别达到52．3万人、84．5万人和64．5万人。城镇10．3万人和农村4万人纳入最低生活保障范围。对11.5万名鞍钢厂办大集体企业长期放假职工实行定期定额救助。为1.9万名“4555”人员落实了社保补助资金。对10余万城乡特困人口开展了临时救助，资助了3万名困难家庭学生就学。为1000名贫困白内障患者实施了手术。棚户区和危房改造全面启动，拆迁改造棚户区27片、31.7万平方米，完成拆迁总量的76.2％。

（九）积极推进依法行政，全面加强政府自身建设。坚持重大事项及时向人大及其常委会报告和与人民政协进行民主协商制度。出台了《鞍山市人民政府办理人大代表建议、政协提案工作规则》，190件人大代表建议和368件政协提案全部办结。首次举办了行政复议听证会和立法听证会。向人大提报立法议案4件，制定、修改、废止政府规章17件。建立了食品安全监管体系，出台了《鞍山市食用农产品安全监督管理办法》，全面启动食用农产品市场准入制度，在市区全面实施了牛羊集中屠宰。规范了小区物业、城市供热和出租车市场管理。打击非法营运出租车专项行动取得阶段性成果。建立了政府决策咨询体系。公共行政服务体系进一步完善。在省内率先推行政府信息公开。全面推进诚信体系建设，鞍山被确定为省诚信体系建设示范城市。强化行政责任追究，全年对213名行政违纪违法人员进行了处分。

各位代表，回顾发展历程，我们所取得的每一个进步，都是全市各族人民同心同德、艰苦奋斗和社会各界支持帮助的结果。在此，我代表鞍山市人民政府，向辛勤工作在各条战线上的全市人民，向为鞍山做出重大贡献的鞍钢等中省直单位，向驻鞍部队和武警官兵，向给予政府工作大力支持和有效监督的人大代表、政协委员、民主党派、工商联、各人民团体和社会各界人士，以及所有关心支持鞍山发展的海内外朋友，致以崇高的敬意和衷心的感谢！

各位代表，在总结成绩的同时，我们也清醒地认识到工作中还仍然存在着一些问题。主要是资源浪费和生态环境破坏现象比较突出；对外开放领域不宽，利用外资规模不大，投资环境还需进一步优化；农村社会事业发展相对滞后，农民增收比较缓慢；劳动就业和社会保障压力加大，部分城乡群众生活还比较困难；政府职能转变还不到位，一些政府工作人员服务意识不强，工作作风还有待于进一步改进。对上述问题，我们要在今后的工作中认真加以解决。

二、“十一五”主要任务和2006年政府重点工作

在市委的领导下，市政府组织编制了《鞍山市国民经济和社会发展第十一个五年规划纲要（草案）》，提请本次会议审议。

“十一五”时期，全市经济社会发展的指导思想是：以邓小平理论和“三个代表”重要思想为指导，认真贯彻党的十六大和十六届五中全会精神，坚持以科学发展观统领经济社会发展全局，坚持科学发展鞍山，突出振兴老工业基地、建设和谐鞍山主题，强化工业强市方略，加快改革开放，增强自主创新能力，推进经济结构调整，转变经济增长方式，提高经济运行质量和效益，统筹城乡发展，提高人民生活水平，提升城市综合竞争力，促进经济持续快速协调健康发展和社会全面进步，率先实现鞍山老工业基地全面振兴，努力开创鞍山经济社会发展的新局面。

今后五年，全市经济增长速度和效益增幅要达到或超过东部地区平均水平，城市居民人均可支配收入增幅要达到或超过沿海省份平均水平，农民人均纯收入增幅要达到或超过东部地区平均水平，实现“总量倍增、位次前移、追赶沈大、殷实和谐”的目标，进入全省第一集团，成为振兴辽宁的重要支撑。

按照上述的指导思想和总体目标，“十一五”时期全市经济社会发展的主要任务是：

――实现经济总量倍增，综合经济实力在国内位次前移。地区生产总值年均递增13％以上；2010年人均地区生产总值比2000年翻两番；万元地区生产总值能源消耗比“十五”末降低25％以上；地方财政一般预算收入增长高于经济增长；全社会固定资产投资年均递增20％以上。在中国城市综合实力百强排名中由2005年的第44位跃升至前40位。

――产业结构进一步优化，核心竞争力不断提升。加快构筑“一个基地、三大产业”格局，扶持企业争创中国名牌产品和中国驰名商标。加快城乡一体化进程，努力建设社会主义新农村。大力发展现代服务业。到2010年，形成以高新技术产业为先导，工业为主体，服务业全面发展，现代农业为基础的三次产业协调发展的产业格局。主要经济和社会发展指标追赶沈大，缩小与沈大差距。

――投资环境明显改善，对外吸引力逐步提升。积极融入区域经济一体化发展，使鞍山成为辽宁中部城市群和滨海经济带之间的重要隆起带和连接带。基本建立起完善的公共行政服务体系和诚信体系框架。进入国家环保模范城市和国家生态园林城市行列。到2010年，建成区空气环境质量全年达到国家二级标准天数80％以上。五年实际利用外资年均递增20％，外贸出口总额年均递增15％。

――体制机制更加完善，发展活力充分释放。积极转变政府职能。市场在资源配置中的基础性地位基本确立，形成有利于全民创业、促进经济全面协调可持续发展的体制机制。非公有制经济保持年均15％增长速度，到2010年，占全市经济总量比重超过60％。

――社会事业全面进步，城市凝聚力明显增强。全市实现普及高中阶段教育。社会保险基本实现全覆盖。五年安置就业50万人次。城镇登记失业率控制在4．5％以内。城市化率达到65％以上。城市居民人均可支配收入和农民人均纯收入年均分别增长10％以上。全面完成棚户区和危房改造任务。建成数字化电视网络。进入国际健康城市和全国文明城市行列。

2006年是“十一五”开局之年。经过“十五”的发展，鞍山已经具备良好的发展基础和振兴条件，实现经济隆起大有希望。同时也面临着巨大的压力和挑战。我们要想在“十一五”期间实现三个“达到或超过”和追赶沈大，只有以更大的魄力、更快的速度、更好的效益，才能实现所提出的发展目标。“十五”时期，鞍山的快速发展主要得益于鞍钢的跨越式发展，到“十一五”时期，鞍钢将进入稳定的发展期，只有在继续支持鞍钢等国有企业发展的同时，大力发展外资经济、民营经济和县域经济，我们才能完成全市2006年及“十一五”时期的各项任务和奋斗目标。

新的一年，我们将按照市委九届十一次全会暨全市经济工作会议的总体安排和部署，围绕“十一五”规划纲要确定的指导思想、奋斗目标和主要任务，坚持科学发展鞍山，大力实施工业强市方略，以开放促改革、促调整、促发展、促振兴，以“项目、实事、专项工作”为载体，认真落实好各项工作。按照“三高两低一稳定”的总体要求，实现经济社会发展高起点开局，高质量运行，高效益增长，降低消耗和降低失业率，保持社会基本稳定，确保“十一五”开局起步之年鞍山经济社会又快又好发展。主要预期目标是：地区生产总值比上年增长13％以上；地方财政一般预算收入增长14％以上；万元地区生产总值能耗、水耗分别降低5.2％以上；城镇登记失业率控制在5％以下；全社会固定资产投资增长30％以上；城市居民人均可支配收入和农民人均纯收入分别增长10％以上。

按照上述预期目标，我们要重点做好以下十个方面工作：

（一）以项目为核心，促进工业结构优化升级。一是推进精品钢材基地项目建设。继续支持鞍钢发展，实现地企融合，共兴共赢。支持鞍钢东部1100万吨钢现代化改造和西部500万吨精品钢材基地投产达产。支持三冶公司30万吨直缝焊管等33个项目建设。二是推进三大产业项目建设。以鞍钢重机为龙头，推进中油天宝挖掘机等48个重点项目，促进海城永兴实业有限公司液压动力钳等6个项目竣工投产；重点支持海城纺织工业园化纤厂、鞍轮集团年产120万套子午线轮胎等49个项目建设，促进岫岩永安有限公司年产12000吨滑子蘑系列产品等9个项目竣工投产；支持鞍千矿业300万吨精矿粉生产线二期工程等25个重点项目建设，促进海城牌楼镁矿公司高档镁砂等4个项目竣工投产。三是全面促进工业升级。调整完善工业发展空间布局，解决好城区工业发展用地问题。加速“壮二活三”进程，按照工业园区的产业规划，推进城区企业异地搬迁改造。调整优化工业结构，推进企业合资重组、改造升级，促进企业做大做强。落实《品牌立市、名牌兴市工作实施方案》，培育一批拥有自主知识产权的名牌产品和具有核心竞争力的优势企业。

（二）以发展县域经济为重点，扎实推进社会主义新农村建设。一是落实《城乡一体化工作实施方案》。按照“生产发展、生活宽裕、乡风文明、村容整洁、管理民主”的目标要求，制定社会主义新农村建设规划，按地域分布和经济社会发展不同情况，分类分批扎实有效地抓好试点工作。深入开展创建“文明村镇”、“文明户”活动，加强对农民的培训，搞好科学普及和民主法制宣传，提高农民整体素质。二是继续深化农村综合改革。落实党在农村的各项政策，及时足额发放三项补贴资金。完善土地流转制度。推进集体林产权制度改革和动物卫生监督管理体制改革。适时启动城乡统一的新型户籍管理制度。探索建立失地农民的养老和医疗保险制度。三是大力推进农村工业化。引导农村工业企业向园区集中，形成集群效应。发展特色农副产品精深加工业，积极扶持县域骨干企业，培育县乡支柱产业。引导农村劳动力向二、三产业转移。海城市以矿产品深加工、钢铁深加工、轻纺工业为主要方向，台安县以石油化工、农产品深加工为主要方向，岫岩县以矿产品深加工、特色农产品和林产品加工为主要方向，千山区以先进装备制造、产业配套为主要方向，发展产业集群，提高工业化水平。四是推进农业产业化。市财政安排2000万元支持产业化重点项目。启动汤岗子农业高新技术产业基地建设。推进龙兴奶业等11个产业化项目，大力发展规模以上龙头企业。积极发展农民经济合作组织。由市政府出资，在全市每个乡镇设立特色农业技术推广站。引进推广农业新技术。推进市级农村科技示范基地建设。完善市场准入制度，扩大食用农产品市场准入范围。以优质稻米、鲜细菜、高油大豆、南果梨、食用菌、肉用牛羊、肉蛋鸡等特色产品为主，大力发展无公害农产品和绿色食品生产。推进标准化养殖小区建设。增加财政投入和利用国家开发银行贷款，加强农业基础设施建设。五是推进农村城镇化。搞好小城镇规划和建设。加快村镇住宅建设和供水、排水等基础设施建设。改扩建西柳客运东站和台安客运站。新建改建农村公路网412公里。基本实现村村通客车。六是全面改善农村环境。推进清垃圾、清路障、清柴垛、改水、改灶、改厕和村屯绿化等工作，全面加强镇村环境综合治理。推广利用新能源，生物质燃气、沼气和太阳能等清洁能源使用率达到15％以上，比上年提高7个百分点以上。

（三）以推进服务业升级为突破口，大力发展第三产业。一是发展壮大旅游业。办好中国森林旅游博览会暨第六届中国鞍山·千山国际旅游节和第三届中国·鞍山宝玉石博览会等活动。抓住沈阳举办世界园艺博览会的机遇，拓展客源市场。加强东北“4＋1”城市区域旅游联合体合作。积极发展县域旅游和农事旅游。全年实现接待境内外游客突破880万人次，旅游综合收入比上年增长15％以上。二是加快发展现代商贸物流业。调整完善商业发展空间布局，建设多层次城乡商业网络，打造辽宁中南部地区商贸物流中心。积极推进二十一世纪购物广场等22个重大项目建设。促进商业企业对外合资合作。加快铁西人民路、立山广场、湖南和深沟寺等商业副中心建设。在城市中心区主干道两侧逐步实施“退居进商”，推进胜利路两侧商业带开发，积极推进铁西温州国际商品城和兴业广场建设。深入推进“万村千乡”市场工程。建设海城西柳物流配送中心，提高西柳服装批发市场、南台箱包市场的档次和水平。三是积极发展金融等服务业。落实《搞活金融及解决融资难问题工作实施方案》，大力改善金融环境。积极与国家开发银行合作，完善开行软贷款平台。推进市商业银行改制重组。继续推进信用社区试点工作。发展各类融资担保机构。做好企业上市培育工作。继续防范、化解金融风险。大力发展保险、会计、咨询、中介等现代服务业。

（四）以招商引资为先导，进一步推进对外开放。一是大力强化招商引资工作。落实《关于进一步扩大对外开放的若干意见》和《提升城市对外吸引力工作实施方案》。办好中国鞍山（香港）推介会和韩国投资说明会，争办2006年辽宁台湾周活动。围绕拉长产业链和发展产业集群，谋划招商主题，拓展招商领域，创新招商方式，实现全方位、宽领域、多层次招商，提高招商成功率。积极吸引世界500强企业入驻鞍山。全年实际利用外资比上年增长25％以上。二是积极推进对外经贸合作。支持企业开展国际贸易，促进出口基地建设。整顿矿产品出口市场秩序，严格限制镁砂和滑石等初级产品出口。积极引导企业实施“走出去”战略，推进三冶蒙古铁矿等10个外经项目建设。全年对外工程承包劳务合同额实现5500万美元，实现对外劳务输出5000人。三是加强区域经济合作。落实《辽宁中部城市群（沈阳经济区）合作协议》，办好辽宁中部城市群2006年市长联席会议，在基础设施建设、产业配套发展和建立共同市场等方面积极开展合作，主动参与辽宁装备制造业基地产业分工。借助大连港、营口港建设，大力发展临港经济和现代物流业。

（五）以深化各项改革为着力点，继续推进体制机制创新。一是继续深化国有企业改革。重点是逐步解决国有企业和已改制企业的历史遗留问题、职工的社会保障和再就业问题。做好改制破产企业职工安置、保险接续和内欠偿还、档案管理等后续工作。二是加速全市集体企业改制步伐。做好集体企业改革试点工作。落实《促进全市厂办大集体企业改制分流人员再就业工作实施方案》。三是进一步优化非公有制经济发展环境。落实《创造适合非公有制经济发展环境工作实施方案》。继续完善促进非公有制经济发展的政策措施，鼓励和支持全民创业。用好支持非公有制经济发展专项资金。全市非公有制经济主要指标增长15％以上。四是积极推进财税体制改革。全面实施国库集中收付制度改革。巩固农村税费改革成果，进一步减轻农民负担。依法加大农业、社保、基础教育、公共医疗、特困群体等方面资金投入。五是强化国有资产管理。对全市国有资产实行统一管理、规范运作和全方位监督，实现国有资产保值增值，防止国有资产流失。

（六）以科技创新为动力，深入实施科教兴市和人才强市战略。一是加速发展高新技术产业。全面增强自主创新能力，提升产业整体技术水平。落实《增强科技自主创新能力工作实施方案》。建立以企业为主体、市场为导向的技术创新体系。大力支持科研院所、企业技术中心的发展，坚持集中化研究、规模化生产，加速科研成果产业化，加强原始创新、集成创新和引进消化吸收再创新能力。注重用高新技术改造传统产业，以信息化带动工业化。办好海外学子活动周。继续推进校企合作。发展壮大拥有自主知识产权的企业和高新技术企业。二是加快高新区建设。调整完善高新区发展规划，拓展发展空间，加大基础设施建设力度。充分利用园区的政策和技术优势，大力发展机电一体化、装备制造等高新技术产业。高新区全年实现地区生产总值比上年增长35％以上。三是大力发展教育事业。落实《维护教育公平、普及高中阶段教育、提高高等教育水平工作实施方案》。高度重视发展高等教育。加强基础教育，深化素质教育，全面推进“双高普九”。新建6所农村九年一贯制学校，发展22所高级特色学校。依法规范民办教育。加强职业技术教育，加大城乡职业教育资源整合力度，提高办学水平。四是落实人才强市战略。落实党的人才政策，注重发挥人才作用。进一步完善用人机制，重奖有突出贡献的拔尖人才，充分调动和发挥各级各类人才的积极性。加强博士后工作站、民企博士后科研基地和引智示范基地建设，积极引进国内外各类人才。重视大中专毕业生的就业工作。

（七）以城乡建设和管理为支撑，加快建设资源节约型、环境友好型社会。一是加强城市规划和基础设施建设。落实《拓展城市空间和区划调整工作实施方案》，修编城市总体规划，调整土地利用总体规划，搞好功能区详细规划，调整和完善工业和服务业发展布局，解决城区发展空间不足问题。强化对土地交易的统一管理。完成汤岗子组团和千山组团开发控制性详细规划，并适时启动建设。规划建设鞍山西部等3个500千伏和海城英落等3个220千伏变电所。开工建设引汤入鞍二期、城市集中供热和第二污水处理厂二期等城建打捆项目。启动海岫丹高速公路（鞍山段）建设。完成鞍腾路、园林路、解放路部分路段和陈家台路等道路大修改造工程。新建建材街道路、深营路东线隧道、千山中路科大过街天桥。二是加强资源保护和合理开发。落实《建设节约型社会工作实施方案》。重点抓好矿产资源的合理开发利用和矿产品的精深加工，深加工比例提升5个百分点以上。全面整顿和规范矿产资源开发秩序，取缔无证勘察开采，坚决制止乱采滥挖、浪费破坏矿产资源、严重污染环境等违法行为，严肃查处越界开采、非法转让探矿权等行为。合理开发利用水资源，推广节水先进技术和工艺，加强自备水井的统一管理。三是加强生态环境保护和污染治理。落实《建设环境友好型社会和绿色鞍山工作实施方案》、《完善城市防洪设施工作实施方案》。深入推进国家环保模范城创建活动。植树造林6万亩，植树2000万株。突出街角地头的绿化美化，见缝插绿，拆墙透绿。新植25条道路的行道树，新建道路基础绿地11万平方米，居民小区绿化及小游园建设10万平方米。完成鞍钢化工总厂三回收系统改造等11项大气环境污染治理工程，整治电厂等燃煤企业二氧化硫污染，控制尾矿库、操场、道路、裸露地面和建筑工地扬尘，建成区空气环境质量全年80％天数达到国家二级标准。继续清理小选矿企业。完成鞍钢西部污水处理厂深度处理等4项水环境治理工程。综合整治机动车尾气、噪声和油烟污染。继续实施矿山生态恢复工程，完成前峪尾矿库、齐大山矿区和眼前山矿区的植被恢复200公顷。依法加大对占道经营、露天烧烤的整治力度。着力解决城乡结合部和部分社区卫生脏乱差问题。

（八）以构建和谐鞍山为主题，促进社会全面进步。一是启动全国文明城市创建工作。大力加强公民道德建设，深入开展群众性精神文明创建活动，积极创建廉洁高效的政务环境、公正公平的法制环境、规范守信的市场环境、健康向上的人文环境、安居乐业的生活环境和可持续发展的生态环境，进一步提高公民思想道德素质和科学文化素质。二是加快发展文化、卫生、体育等社会事业。加快搞好市博物馆建设和群众艺术馆搬迁改造。开展大型文化广场系列活动。加快推进数字化电视网络建设。巩固国家卫生城市成果。启动国际健康城市创建工作。完善市紧急医疗救援系统，扩建市中心医院病房，改造汤岗子医院治疗中心。整合区域卫生资源，组建精神病康复中心、老年病医院和结核病防治院。完善社区医疗设施，搞好社区医疗服务和保健。强化公共卫生防控体系建设，提高应急处置能力。由政府出资，建立和完善县乡兽医服务体系。综合防治艾滋病、流行性出血热、人禽流感等传染病，确保全市无重大传染病疫情暴发。规划和推进新体育场建设，广泛开展全民健身运动，提高竞技体育水平。三是加强社区建设。以便民利民为宗旨，积极发展社区服务网点，全面拓展服务功能。夯实社区“一站四室”管理工作。争创全国社区建设示范市。四是全力做好维护社会稳定工作。进一步完善信访工作责任体系，强化市级领导接访群众制度，建立健全矛盾纠纷排查调处机制，积极有效化解社会矛盾，避免发生影响重大的群体性事件。加强安全监管，防止重特大安全事故发生。深入开展“五五”普法活动，全面建设法治鞍山。继续落实社会治安综合治理的各项措施，加强社会治安防控体系建设，提高防范打击各种犯罪活动和处置各类突发事件的能力，打造“平安钢都”，争创社会治安综合治理先进市。积极创建国家畅通工程绿色交通示范城市。五是落实党的民族政策，加大对少数民族地区的扶持力度。六是继续开展创建全国“双拥”模范城活动，力争四连冠。深入开展地方支持国防事业建设，部队支持地方经济建设的“双支”活动。重视驻鞍部队后勤保障社会化工作的落实。七是进一步做好人口与计划生育、新闻出版、广播电视、妇女儿童、老龄、民族、宗教、侨务、人防、史志、档案、气象、地震、残疾人事业、民兵预备役等方面工作。认真做好全国农业普查的各项准备工作。

（九）以解决群众最急盼解决的问题为切入点，将振兴发展成果惠及广大人民群众。落实《将振兴发展成果惠及广大人民群众工作实施方案》，从群众最急盼解决的问题入手，切实解决好群众最关心、最直接、最现实的利益问题。一是解决好困难群体再就业问题。完善消除“零就业家庭”的长效机制。继续深化“1＋X”就业模式，通过广开就业渠道，增加就业岗位，大力推进失业人员再就业。二是建立完整的社会保障体系。落实《强化社会保险扩面征缴工作实施方案》，继续扩大养老、失业、医疗等保险覆盖面，做到应保尽保。建立和完善覆盖面更加广泛的社会保障制度，在有困难的城镇国有企业、集体企业中探索建立在职职工和退休职工合作医疗制度。制定和实施低保家庭人员医疗救助实施办法。全面实施新型农村合作医疗制度，提高补助标准，使广大农民群众得到更多的实惠。三是继续实施棚户区和危房改造。严格房屋建筑质量管理，完善配套设施，确保棚户区和危房动迁群众按时回迁。四是切实解决城市居民的供暖、供气和供水问题。落实《鞍山市城市供热管理办法》，加强对供暖企业的管理，保证供暖质量。努力拓展城市气源，加强管道燃气设施建设，解决城市居民供气不足的问题。加强水源管理和保护，进一步改善饮用水水质。五是切实维护职工合法权益。落实最低工资标准，严厉查处克扣劳动者报酬、恶意欠薪和肆意延长劳动时间等违法行为。切实维护农民工合法权益，解决好拖欠农民工工资等问题。六是继续实施南沙河综合整治工程。加快推进南沙河两岸开发建设。七是改善居民生活环境。安装鞍海路、矿工路和鞍千路路灯，大修、粉饰和亮化城市主要桥梁。对千山路和鞍海路进行综合整治，全面提高绿化、亮化和美化水平。改造完善东山和二一九公园、孟泰公园基础设施。推进永乐公园周边住宅和企事业单位的搬迁。规划建设深沟寺眼镜湖公园。加快实施居民小区和街巷路改造及亮化工程。广泛开展创建绿色社区和安静小区活动。每个社区至少有一处安装体育健身器材的健身场所。八是深入开展扶贫帮困工作。制定并实施《鞍山市特困群众社会救助暂行办法》。继续做好为农村困难户建房、修房工作。对特困居民提供社会救助。积极探索改善低保家庭中丧失劳动能力和高龄特困人群的生活救助办法。解决困难家庭子女就学问题，从2006年开始，两年内全部免除农村义务教育阶段学杂费。继续对农村贫困家庭学生提供免费课本和寄宿生活费补助，残疾人和低保家庭子女全部免费就学。全面实施高中阶段家庭困难学生助学金资助计划，对低保家庭考入大学的子女，入学时每人一次性资助3000元。九是解决城乡居民看病难、看病贵问题。安排财政资金950万元，用于城乡群众重大疾病医疗救助。设立8家救助定点医院，对未参加医疗保险的城市低保对象实施门诊和住院即时救助。十是解决达道湾污水灌溉水稻问题。调整土地利用规划，逐步将农业用地改为工业用地。同时加快利用污水处理厂中水灌溉农田的进程。

（十）以建设法治政府为目标，加强政府自身建设。一是自觉接受市人大及其常委会的法律监督、工作监督和市政协民主监督，加强与各民主党派及社会各界的联系，主动接受社会监督和舆论监督。完善向人大及其常委会报告工作制度和主动与政协协商制度。落实人大及其常委会的各项决议。将人大代表建议和政协提案办理工作纳入政府重要议事日程和市直机关领导班子目标管理考核体系，对重要的代表建议和政协提案列入市政府常务会议或业务会议研究。推进政府信息公开，增强政府工作的透明度和公众参与度。二是进一步提高依法行政水平。深入贯彻《国务院全面推进依法行政实施纲要》和《鞍山市依法行政五年规划》，推进法治政府建设进程。严格按照法律规定的权限和程序行使政府权力，履行政府职责，协调各方利益关系，促进社会公平和正义，维护社会秩序和稳定。加快电子政务建设，提升政府信息网的综合应用水平。完善政府决策咨询体系，健全重大事项社会公示制度、听证制度和专家咨询制度。完善决策跟踪反馈和责任追究制度。三是深化行政管理体制改革。以转变政府职能为重点，继续推进政企分开，努力增强政府驾驭经济工作、管理社会事务和处理突发事件的能力。全力推进诚信体系建设。四是切实加强政风建设。坚持科学发展鞍山，坚持以人为本、执政为民。要满怀深厚的感情，把解决好人民群众急盼解决的问题，作为政府义不容辞的责任；把解决好人民群众关注的热点、难点问题，作为政府的主要任务；把人民群众的幸福快乐，作为政府的不懈追求。认真贯彻执行《建立健全教育、制度、监督并重的惩治和预防腐败体系实施纲要》，落实好业务目标管理责任制和党风廉政建设责任制。强化政府采购中心建设，完善工程招投标和土地、产权交易制度建设，严格财政资金监督和管理。进一步强化行政监察和审计工作。全面贯彻实施《公务员法》，努力建设一支政治合格、业务精通、清正廉洁、作风优良的公务员队伍。在全市政府机关大力倡导求真务实之风、廉洁从政之风、艰苦奋斗之风，围绕建设勤政、廉政、务实、高效政府的目标，开创政府工作新局面。

各位代表，未来五年的宏伟蓝图已经绘就，面对新的征程、新的机遇、新的挑战，我们一定要坚持以科学发展观为统领，在市委的领导下，在人大和政协的监督支持下，同心同德，埋头苦干，锐意进取，开拓创新，为实现老工业基地全面振兴和建设和谐鞍山而努力奋斗！
